# Targeting of the AXL receptor tyrosine kinase by small molecule inhibitor leads to AXL cell surface accumulation by impairing the ubiquitin-dependent receptor degradation

**DOI:** 10.1186/s12964-019-0377-8

**Published:** 2019-06-06

**Authors:** Markus Lauter, Anja Weber, Robert Torka

**Affiliations:** Institute of Physiological Chemistry, University Halle-Wittenberg, Medical Faculty, Hollystrasse 1, 06114 Halle (Saale), Germany

**Keywords:** RTK, AXL, TKI, Ubiquitin, Degradation, 3D spheroid

## Abstract

**Background:**

Overexpression of AXL receptor tyrosine kinase (AXL) in various human cancers correlates with reduced patients overall survival and resistance to first line therapies. Therefore, several AXL tyrosine kinase inhibitors (TKIs) are currently under clinical evaluation.

**Results:**

AXL TKI BMS777607 treatment increased AXL protein levels after 24 h as observed by Western blot and flow cytometry analysis. Mechanistically, this inhibition-induced AXL cell surface accumulation was neither associated with epigenetic modifications, nor altered transcriptional and translational regulation. Further, we saw no impact on glycosylation and receptor shedding by α-secretases. However, we observed that BMS777607 increased the glycosylated 140 kDa AXL protein abundance, which was impaired in the kinase dead mutant AXL (K567R). We demonstrated that AXL kinase activity and subsequent kinase phosphorylation is necessary for GAS6-dependent receptor internalization and degradation. Blocking of kinase function by BMS777607 resulted in ubiquitination prohibition, impaired internalization and subsequent cell surface accumulation. Subsequently, AXL cell surface accumulation was accompanied by increased proliferation of 3D-Speroids induced by low μM levels of BMS777607 treatment.

**Conclusion:**

Our data suggest a re-evaluation of anti-AXL clinical protocols due to possible feedback loops and resistance formation to targeted AXL therapy. An alternative strategy to circumvent feedback loops for AXL targeting therapies may exist in linkage of AXL TKIs to a degradation machinery recruiting unit, as already demonstrated with PROTACs for EGFR, HER2, and c-Met. This might result in a sustained inhibition and depletion of the AXL from tumor cell surface and enhance the efficacy of targeted anti-AXL therapies in the clinic.

## Background

Deregulated oncogenic activity of the AXL receptor tyrosine kinase (AXL) and elevated levels of its ligand GAS6 (growth arrest specific gene 6) are found in numerous types of human cancer and are directly correlated with diverse aspects of cancer pathogenesis [1]. AXL up-regulation has also been described in cisplatin-resistant ovarian cancer, doxorubicin-resistant acute myeloid leukemia, lapatinib-resistant breast cancer, imatinib-resistant gastrointestinal stromal tumors, and imatinib-resistant chronic myeloid leukemia. Recently, AXL activation has been reported as a cause of resistance to epidermal growth factor receptor (EGFR)-targeted therapy in non–small cell lung cancers [2, 3].

AXL expression is regulated via various mechanisms. It is subject to epigenetic modifications, i.e. DNA methylation and histone acetylation [4]. Hypomethylation of the AXL gene has been associated with high expression in different cancer entities. Multiple transcription factors, including SP1, AP-1 and HIFα, can induce AXL transcription [5, 6]. Expression of splice variants is regulated post-transcriptionally and by translation initiation factors, e.g. eIF4E and eIF4B [7]. AXL undergoes either N-linked or O-linked glycosylation in the Golgi apparatus before it is properly recruited to the membrane [8, 9]. AXL kinase activity is increased after GAS6 binding leading to activation of downstream signaling cascades [4]. AXL signaling stimulates phosphatidylinositide 3-kinase/RAC-α serine/threonine protein kinase (PI3K/AKT), extracellular signal-regulated kinase (ERK) and p38 mitogen-activated protein kinase cascades (MAPK), the nuclear factor-kappa B (NF-κB) pathway as well as signal transducer and activator of transcription signaling (STAT) [10]. Therefore, biological processes including invasion, angiogenesis, resistance to chemotherapeutics and targeted drugs, survival, and proliferation, as well as receptor downregulation are tightly regulated. Phosphorylated AXL is internalized for subsequent degradation or recycling via proteasomal, lysosomal or endosomal pathways [11–13]. Since deregulated AXL expression is associated with cancer and other pathological conditions, a better understanding of AXL regulation is critical for AXL-targeted treatment approaches. A complex of γ-carboxylated GAS6 Ca^2+^-dependently bound to phospholipid PtdSer causes homo-dimerization of the receptor, leading to the optimal activation of AXL kinase by phosphorylation of tyrosine residues at the receptor C-terminal kinase domain. This phosphorylation event creates docking sites for AXL downstream signaling molecules [14, 15]. In parallel, AXL phosphorylation leads to recruitment of the GRB2 adaptor protein, which recruits the E3 ubiquitin-protein ligase CBL in similar fashion as shown for c-MET [16, 17]. An additional mechanism is the direct recruitment and activation of CBL through the CBL tyrosine kinase binding (TKB) domain. Upon binding and activation of CBL, AXL is ubiquitinated [18]. This posttranslational modification is required for efficient AXL degradation in the lysosome [13]. An alternative pathway of AXL degradation is the proteolytic shedding by ADAM10 and ADAM17, as described by Miller et al., releasing a soluble 85 kDa N-terminal fragment (sAXL) and a short intracellular 55 kDa C-terminal fragment [19]. This soluble sAXL receptor can be used as a biomarker and companion diagnostic tool as shown for hepatocellular carcinoma (HCC) and malignant peripheral nerve sheath tumors (MPNST) [20, 21]. Because AXL is considered an important molecular target in cancer therapy, various strategies, like sAXL decoy receptor (GL2I.T and MYD1–72) and antagonistic monoclonal antibody targeting AXL (YW327.6S2 and 20G7-D9) are currently in preclinical development. Other applications have already proceeded to clinical trials [22]. Among the most promising AXL-inhibitory approaches are small molecule tyrosine kinase inhibitors (TKIs). In the present study we used the preclinical AXL/MET small molecule inhibitor BMS777607 (BMS) [23]. Here we show that AXL inhibition by BMS causes accumulation of the AXL receptor on the cellular surface due to impaired receptor downregulation. We propose that inhibition of AXL kinase phosphorylation by the small molecule inhibitors causes reduced ubiquitination of AXL leading to reduced lysosomal degradation. This might be linked to therapy resistance displaying a major problem of targeted cancer therapies in the clinic. This study elucidates the complex regulation of AXL expression.

## Methods

### Cell culture

Cells were cultured in a humidified incubator with 5% CO_2_ at 37 °C. MDA-MB231 was maintained in DMEM supplemented with 1% sodium pyruvate and 10% FCS. MDA-MB231-D3H2LN (Caliper) [24] was grown in MEM supplemented with 1% sodium pyruvate, 1% GlutaMAX, 1% nonessential amino acids, and 10% FCS. Hs578T and NCI-H292 (H292) and NCI -1792 (H1792) were cultured in RPMI 1640 medium with 1% GlutaMAX and 10% FCS. Cell culture media and supplements were purchased from Gibco (Thermo Fisher Scientific, Darmstadt, Germany).

### Three-dimensional (3D) spheroid culture and cell viability

Matrigel basement membrane matrix (BD Biosciences—Corning, No. 354234; Kaiserslautern, Germany) was diluted to a concentration of 3 mg·mL^− 1^ in cell line-corresponding serum-free medium. 7000 cells were seeded on top of the solid Matrigel in each well (96-well plate). Viability of cells was determined by ATP quantification using CellTiter-Glo Luminescent Cell Viability Assay (Promega, Mannheim, Germany). The luminescence signal was measured by Clariostar multimode microplate reader (BMG Labtech, Ortenberg, Germany).

### Tyrosine kinase inhibitors and cell treatment

BMS777607 was purchased from ShangHai Biochempartner Co., Limited, Wuhan, China (Cas No.:1196681–44-3) with a purity > 98%. Chloroquine Phosphate (CQ, No.S4157 in H_2_O 10 mg/ml) and DAPT were purchased from Seleckchem (No. S2215, BIOZOL GmbH, Eching, Germany). BB94/batimastat (BB94, No. 2961) and human recombinant GAS6 (#885-GSB-050) were purchased from R&D Systems GmbH. Cycloheximide (No. C1988) was purchased from Sigma Aldrich. All inhibitors were dissolved in DMSO (Sigma Aldrich, Taufkirchen, Germany), except CQ, and stored at room temperature in 10 mM stock solutions.

### Reverse transcription-quantitative PCR (RT-qPCR)

Gene expression was analyzed following the isolation of total RNA using the RNeasy minikit (Qiagen, Hilden, Germany) and cDNA using random hexamers and Verso cDNA synthesis kit (Thermo Fisher Scientific, Darmstadt, Germany) according to the manufacturer’s instructions. RT-qPCR was performed with DyNAmo ColorFlash SYBR green qPCR kit (Thermo Fisher Scientific, Darmstadt, Germany) using a LightCycler 480 II (Roche, Mannheim, Germany). Relative gene expression levels were calculated according to the 1.9 ^-Δ(CT(housekeeping)-CT(gene of interest)^. Primer sequences: ALAS1, forward: 5′-CTGCAAAGATCTGACCCCTC-3′, reverse: 5′-CCTCATCCACGAAGGTGATT-3′, Human GAPDH, forward: 5′-ACCCAGAAGACTGTGGATGG-3′, reverse: 5′-TTCTAGACGGCAGGTCAGGT-3′, AXL P3. forward: 5′-GAGGGAGAGTTTGGAGCTGT-3′, reverse: 5′-TCATGACGTTGGGATGGTCA-3′, AXL PB, forward: 5′-CAGCTTCGGCTAGGCAG-3′, reverse: 5′-TCCGCGTAGCACTAATGTTCT-3′.

### Western blot analysis

Standard mini gel electrophoresis and blotting system was used (Bio-Rad Laboratories GmbH, München, Germany). Proteins were transferred to PVDF membranes and incubated overnight at 4 °C with the primary antibody. Anti- HA-Tag (No.3724), GAPDH (No.5174), AXL (No.8661) were purchased from Cell Signalling Technonoly (New England Biolabs GmbH, Frankfurt am Main, Germany), and AXL H-3 (No.sc-166,269) from Santa Cruz Biotechnology as well as β-ACTIN from Sigma. Species corresponding fluorophore-labeled secondary antibodies (Li-Cor, Lincoln, NE) were incubated for 1 h at room temperature. The fluorescence signals were detected with an Odyssey CLx system and quantified by Image Studio software (Li-Cor). All bands were normalized as described to ACTIN or GAPDH as loading control.

### Immunoprecipitation

Immunoprecipitation was performed as previously described [25]. The FK-2 antibody (No. BML-PW8810–0100, Enzo Life Sciences GmbH, Lörrach, Germany) was used for ubiquitin precipitation in the concentration of 1 μg per 750 μg lysate. Subsequently, the AXL antibody (No.8661, Cell Signaling, New England Biolabs GmbH, Frankfurt am Main, Germany) was used for western blot analysis.

### Flow cytometry

Cells were detached with Accutase (No. A1110501, Thermo Fisher Scientific, Darmstadt, Germany). Centrifugation was performed at 300 g for 3 min. Cells were fixed with 3.7% PFA at 37 °C for 10 min and permeabilized with − 20 °C methanol for 5 min. Primary antibody Ab 154 (MAB154, R&D Systems GmbH) or Ab 259/2 homemade antibody (clone 259/2, IgG1 isotype) was incubated for 1 h and subsequently incubated for 30 min at 37 °C with Alexa Fluor 488-conjugated secondary antibody (Jackson ImmunoResearch Europe Ltd., Cambridgeshire, UK). Fluorescence intensity was measured using a BD Acccuri Flow Cytometer (BD, Heidelberg, Germany).

### SOMA (single oligonucleotide mutagenesis and cloning approach)

Plasmid for AXL wildtype overexpression with HA-Tag was purchased at Sino Biological (Cat: HG10279-CY). Primer for K567R mutation (GTGGCCCTGGGGAGGACTCTGGGAGAG). SOMA was performed according to Pfirrmann et al. with the following modifications: Q5-polymerase was used in combination with standard PCR protocol [30 cycles 1 min per kb elongation]. DNA was isolated using Qiagen PCR purification kit (Qiagen, Hilden, Germany) [26]. DH5α competent *E. coli* were used for plasmid amplification.

### Statistical data analysis

Mean values and SEM are shown. The statistical analysis was performed by the application of an two-way ANOVA in combination with Bonferroni multiple comparison post-test using GraphPad prism 7 (GraphPad Software, Inc., La Jolla, CA, USA). Differences with *P < 0.05 were considered as statistically significant.

## Results

### Increased AXL protein level after 24 h of low μM treatment with selective AXL TKI BMS777607

**Fig. 1:**
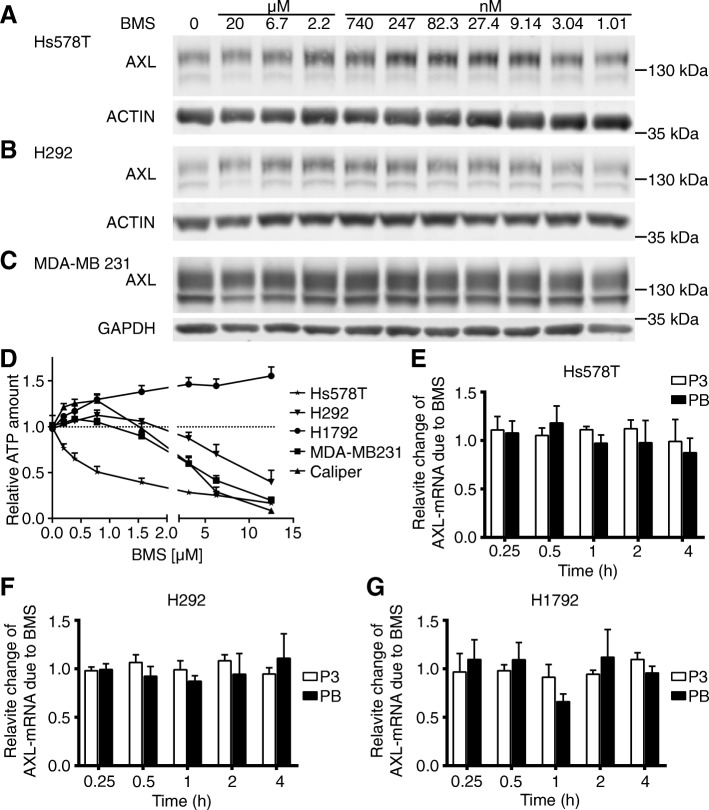
Increased AXL protein level after 24 h of low μM treatment with selective AXL TKI. BMS777607 (BMS) treatment led to increased AXL protein levels in (**a**) Hs578T, (**b**) H292 and (**c**) MDA-MB231 cells. Western blots of AXL after treatment with a three-fold serial dilution of BMS from 20 μM to 1.01 nM are shown. ACTIN or GAPDH served as loading controls. A representative sample of multiple biological replicates is displayed. (**d**) Treatment with low μM concentrations of BMS increased the viability of 3D cell spheroids. Cells were cultivated in a 3D matrix in the presence of BMS (0.001 μM to 12.5 μM). ATP measurements were performed after 72 h. All results were normalized to DMSO control values as indicated by a doted horizontal line. Low BMS concentrations between 0.097 μM and 0.78 μM increased significantly the ATP content of Caliper, MDA-MB231, H292 and H1792 cells in a rage of 110 to 125%, in contrast to Hs578T cells. BMS treatment had no impact on AXL mRNA transcription. 0.5 μM BMS treatment showed no change of AXL mRNA levels by RT-qPCR analysis within 4 h in (**e**) Hs578T, (**f**) H292 and (**g**) H1792 cells. Housekeeping genes GAPDH and ALAS served as normalization controls utilizing the ΔΔCT method. Two different primer sets were used namely, P3 and PB. Mean values and SEM of three independent experiments are shown

AXL expressing Hs578T, H292 and MDA-MB231 cell lines were treated with AXL TKI BMS777607 (BMS) for 24 h. Subsequently, AXL protein levels were quantified by western blot analysis. AXL protein levels were increased 1.5 to 2-fold by low μM concentrations BMS ranging from 0.009 μM up to 2.2 μM in Hs578T cells (Fig. 1a). Analogous results were obtained for H292 and MDA-MB231 cells (Fig. 1b-c). The increase of AXL protein level after treatment with selective AXL TKI BMS is a general phenomenon and correlates with the IC_50_ concentration of BMS for AXL phosphorylation, ranging from 0.005 μM to 0.17 μM (Hs578T, MDA-MB231, BT549), as published by Penzes et al. and Torka et .al. [23, 25].

### Increased viability of tumor spheroids upon low μM treatment with BMS777607

We performed ATP measurements after 72 h of treatment with increasing concentrations of BMS (0.197 μM up to 12.5 μM), to evaluate the impact of increased AXL protein levels on cell viability of 3D-spheroids. Hs578T, MDA-MB231, Caliper, H1792 and H292 were used for spheroid formation and subsequent ATP measurement. Three different cell viability phenotypes were observed. Hs578T cells displayed a concentration-dependent decrease of cell viability. In contrast, H1792 cells showed a concentration-dependent increase of cell viability. The third phenotype was observed in MDA-MB231, Caliper and H292 cell lines, where BMS concentrations higher than 5 μM caused reduced cell viability, whereas concentrations lower than 1 μM led to increased cell viability compared to DMSO- controls, as shown in Fig. 1d. The elevated AXL protein levels correlated with increased cell viability in MDA-MB231, Caliper, H1792 and H292 cell line which might indicate that AXL protein upregulation is part of AXL TKI resistance or feedback mechanism.

### Transcription of AXL mRNA was not affected by low μM treatment with BMS777607

The major mechanism that increases protein concentrations is the transcription of coding mRNA. Therefore, we evaluated the transcription of AXL mRNA in Hs578T, H292 and H1792 cells by RT-qPCR after 0.25, 0.5, 1, 2 and 4 h of treatment with 0.5 μM BMS compared to DMSO control. We observed no significant changes of mRNA levels within 4 h of treatment with BMS (Fig. 1e-g), although a 1.3-fold increase of AXL protein levels was evident already after 3 hours of BMS treatment in H292 cells (Fig. 3e). Based on these results we conclude that low μM BMS treatment does not influence AXL mRNA level. AXL mRNA quantity did not change after 24 h as well (data not shown).

### The significant increase of AXL protein levels by 0.5 μM BMS777607 treatment is depending on culture conditions

**Fig. 2:**
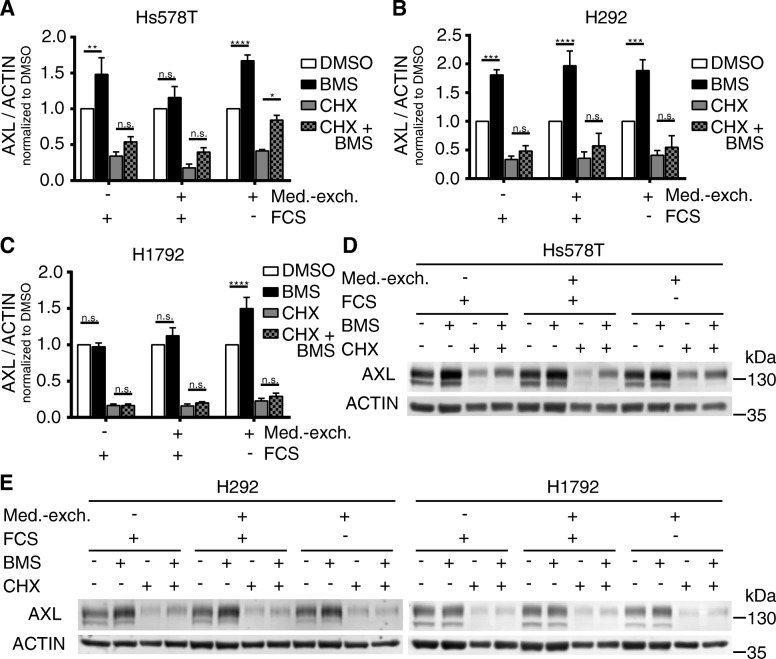
The significant increase of AXL protein levels by 0.5 μM BMS777607 treatment is depending on culture conditions. Combination treatments of cycloheximide (CHX) and BMS777607 (BMS) were performed to discriminate the effects of BMS on translation and proteolysis of AXL. Additionally, we analyzed the impact of serum conditions (+/− FCS) and accumulation of AXL ligand GAS6 by medium exchange prior to the combinatorial CHX and BMS treatment. **a** AXL protein levels were increased significantly by BMS treatment in Hs578T cells. This effect is lost by exchange to serum containing medium. **b** AXL protein levels were increased significantly by BMS treatment independent of cell culture medium conditions or serum concentration in H292 cells. **c** H1792 cell responded to BMS treatment only after replacement of serum containing cell culture medium to serum depleted medium. **d** and **e** Representative western blots of biological replicates are shown. Mean values and SEM of four (H292) or five (Hs578T and H1792) independent experiments are shown. Differences with *P < .05, **P < .01, ***P < .001 and ****P < .0001

We performed combination treatments of Cycloheximide (CHX) and BMS to discriminate between the effects of BMS on translation and proteolysis of AXL protein. Additionally, we analyzed the impact of different cell culture conditions regarding serum concentration (+/−FCS) and accumulation of AXL ligand GAS6, by exchanging the medium prior to the combined CHX and BMS treatment (Fig. 2a-e). It has been shown previously, that GAS6 secretion is stimulated by serum depletion in NIH-3 T3 fibroblasts and leads to ligand accumulation in the supernatant over time [27]. AXL protein levels were increased significantly by BMS treatment independent of cell culture conditions in H292 cells (Fig. 2b). We observed a similar increase of AXL abundance in Hs578T cells as in H292 cells with one exception, when medium was exchanged to fresh serum containing medium (+FCS), which abolished AXL increase (Fig. 2a). H1792 cells responded to BMS treatment only after replacement of serum containing cell culture medium (+FCS) to serum depleted medium (Fig. 2c). CHX at a concentration of 10 μg/ml blocked AXL translation in Hs578T, H292 and H1792. CHX treatment for 24 h had a minimal impact on ACTIN protein level as displayed in Fig. 2d-e. After combined treatment of BMS and CHX we still observed the promoting effect of BMS on AXL protein abundance, although being reduced to a non-significant level. Combinatorial treatment with CHX and BMS resulted in a significant increase of AXL protein levels in Hs578T only after replacement of serum containing cell culture medium to serum depleted medium (Fig. 2a). H1792 responded to serum depleted medium with decreased AXL protein abundance in comparison to the DMSO control without medium exchange (Fig. 2e). Therefore, we concluded that BMS influenced AXL degradation and not protein synthesis. This degradation is not detectable in serum conditions, based on absent secretion of AXL ligand GAS6 in H1792 under these conditions. This is in line with previous reports, that growth arrest specific gene 6 (GAS6) expression and secretion is induced by serum deprivation [27]. These results led us to the hypothesis, that serum depletion and GAS6 accumulation might affect the activation and subsequent degradation of AXL. Furthermore, BMS treatment might impair the degradation of AXL, resulting in an up to two-fold accumulation of AXL protein within after 24 h.

### GAS6 mRNA levels were significantly different in Hs578T, H292 and H1792 cell lines. H1792 cells showed significant induction by serum deprivation

**Fig. 3:**
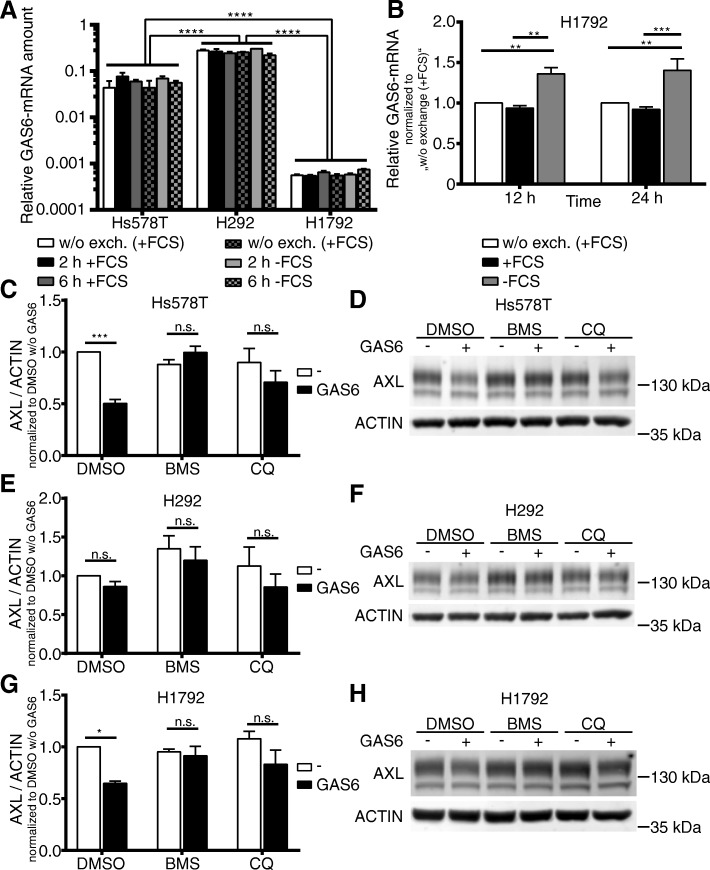
GAS6 mRNA levels were significantly different in Hs578T, H292 and H1792 cells. H1792 cells showed significant induction by serum deprivation. **a** H292 exhibited a 429-fold expression of GAS6 mRNA and Hs578T a 96-fold higher expression compared to H1792 cells. RT-qPCR demonstrated no different expression of GAS6 mRNA in Hs578T, H292 and H1792 cells after medium exchange for 2 hours and 6 hours with serum containing culture medium (+FCS) or serum depleted medium (−FCS). **b** GAS6 mRNA was significantly induced by serum deprivation in H1792 cells after 12 and 24 h of serum depletion. GAPDH and ALAS served as normalization controls utilizing the ΔCT method for (A) and the ΔΔCT method for (B). **c, e, g** Treatment with exogenous GAS6 led to AXL degradation in Hs578T and H1792 cells. BMS777607 (BMS) prohibited lysosomal degradation more efficiently than QC. AXL abundance in Hs578T, H292 and H1792 cells after treatment with AXL TKI BMS or the lysosomal acidification inhibitor Chloroquine (CQ) for 3 hours in combination with 250 ng/ml of recombinant GAS6 for 2 hours is shown. ACTIN served as normalization control. Mean values and SEM of three (H1792), four (H292) and five (Hs578T) independent experiments are shown. **d, f, g** Representative western blots are displayed for Hs578T, H292 and H1792 cells. Differences with *P < .05, **P < .01, and ***P < .001 were considered statistically significant

In order to elucidate the transcription pattern of GAS6 ligand in Hs578T, H292 and H1792 cells, we quantified GAS6 mRNA levels using different serum culture conditions and time points by RT-qPCR. We compared the GAS6 mRNA abundance after cell culture medium exchange and serum deprivation for 2 h and 6 h relative to the abundance of housekeeping genes *alas* and *gapdh*. Cell culturing conditions had no impact on GAS6 mRNA levels in Hs578T and H292 cells (Fig. 3a). Serum deprivation led to a 1.5-fold increase of GAS6 mRNA within 12 h to 24 h in H1792 cells (Fig. 3b). We observed major differences in the GAS6 mRNA abundance between H1792, Hs578T and H292 cells. H1792 cells expressed the lowest amount of GAS6. Hs578T cell displayed a 96-fold higher expression compared to H1792 cells. H292 cells expressed the highest GAS6 mRNA amounts being 4 times higher than in Hs578T und 429 times higher compared to H1792 cell (Fig. 3a). Finally, we hypothesized that GAS6 availability might influence AXL activation and turnover. H292 cells would correspond to a cell type with high GAS6 availability and turnover of the AXL-RTK in contrast to Hs578T and especially H1792 cells. This might explain why BMS impacts in H1792 on AXL expression only after serum deprivation and subsequent induction of GAS6 expression (Fig. 2c, e).

### GAS6-dependent degradation of AXL was prevented by BMS777607 treatment

**Fig. 4:**
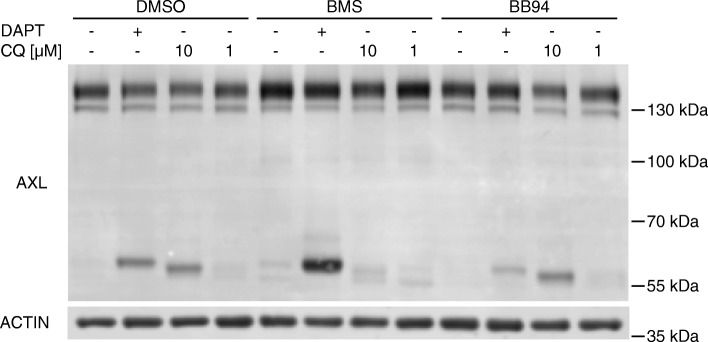
BMS777607 (BMS) is influencing the lysosomal degradation pathway. Hs578T cells were treated with 0.5 μM BMS or 2 μM BB94 in combination with 1 μM DAPT or 1/10 μM CQ for 24 h. DAPT stabilized the 55 kDa C-terminal AXL fragment by inhibition of further degradation by γ-secretases. The α-secretase inhibitor BB94 decreased the abundance of the 55 kDa C-terminal AXL fragment by blocking the AXL receptor shedding. BMS in contrast to BB94 increased the abundance of the 55 kDa C-terminal AXL fragment, indicating that the increased abundance of the 140 kDa AXL is not caused by off-target α-secretase inhibition. 10 μM CQ stabilized a 54 kDa C-terminal AXL fragment by inhibition of lysosomal acidification. BMS reduced the abundance of the 54 kDa C-terminal AXL fragment suggesting that BMS influenced the lysosomal degradation pathway

The impact of GAS6-dependent AXL activation and subsequent degradation was further elucidated by treatment of Hs578T, H292 and H1792 cells with 250 ng/ml recombinant GAS6. GAS6 stimulated cells were pretreated for 1 hour with either AXL TKI BMS or the lysosomal acidification inhibitor Chloroquine (CQ) to prevent lysosomal degradation. The GAS6-dependent degradation was determined by western blot analysis after additional 2 hours of AXL stimulation by supplementing the supernatant with 250 ng/ml GAS6. In H292 cells we observed that recombinant GAS6 could not enhance AXL degradation (Fig. 3e-f), in contrast to Hs578T (Fig. 3c-d) and H1792 cells (Fig. 3g-h), where recombinant GAS6 reduced AXL abundance significantly to 50% in Hs578T and 60% in H1792 cells. This degradation of AXL was completely blocked by 0.5 μM BMS or 10 μM CQ treatment. The effect of BMS was more pronounced when compared to CQ (Fig. 3c, g). Exogenous GAS6-depentent AXL stimulation in combination with relative low endogenous GAS6 expression led to subsequent receptor degradation in Hs578T and H1792 cells. H292 cells, representing a cell line with high endogenous GAS6 expression, did not respond to exogenous GAS6 stimulation. Our result implied that BMS might inhibit the lysosomal degradation pathway of AXL leading to subsequent AXL accumulation. This assumption was further supported by the appearance of a 54 kDa fragment after treatment with 10 μM CQ prohibiting lysosomal degradation of C-terminal AXL fragments in Hs578T cell (Fig. 4). Previous reports described the appearance of a 55 kDa C-terminal fragment after α-secretase cleavage and a 52 kDa fragment after additional cleavage by γ-secretases. These fragments could be stabilized by blocking of γ-secretase by DAPT for the 55 kDa fragment and inhibition of the proteasome with MG132 for the 52 kDa fragment [11]. Additional treatment with 0.5 μM BMS led to an enrichment of the 55 kDa C-terminal fragment stabilized by DAPT and to a decreased abundance of the 54 kDa fragment generated by CQ. This indicated that the lysosomal degradation pathway is affected by BMS (Fig. 4).

### BMS777607 impaired GAS6-dependent internalization of AXL leading to cell surface accumulation of AXL protein

**Fig. 5:**
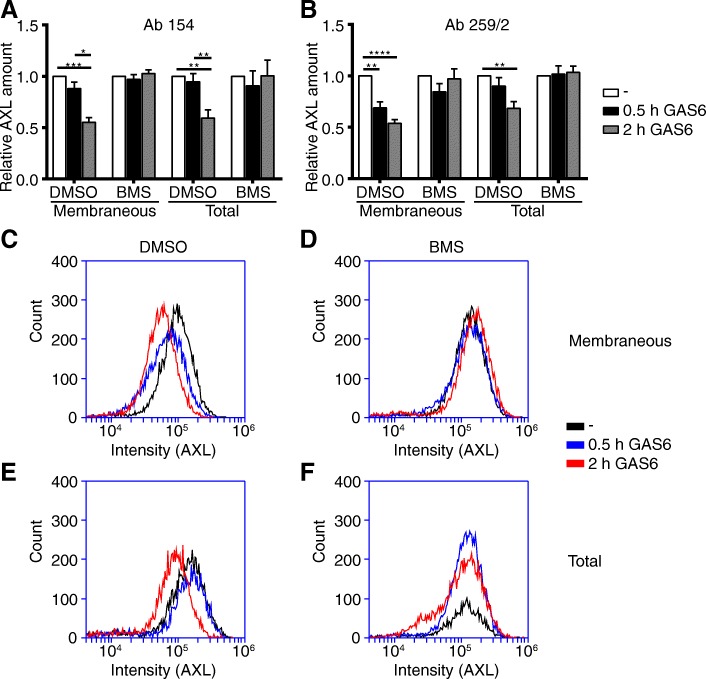
BMS777607 (BMS) impaired GAS6-dependent internalization of AXL. Flow cytometry analysis of Hs578T cells after stimulation of AXL by 250 ng/ml exogenous GAS6 for 30 min and 2 hours in combination with BMS treatment. Two different extracellularly binding anti-AXL antibodies (Ab 154 for **a**, and Ab 259/2 for **b-f**) were used. Membrane permeabilisation was implemented to distinguish between the membranous and total AXL fraction. Representative histogramms of flow cytometry analysis are displayed for Ab 259/2 in the non-permebilized membranious AXL fraction of Hs578T cells (**c-d**) and membrane permebilized total AXL protein fraction (**e-f**). Histogramm C and E represent the DMSO treated Hs578T control cells and D and F show histograms of 0.5 μM BMS treated Hs578T cells. Mean values and SEM of three independent experiments are shown. Differences with *P < .05, **P < .01, ***P < .001 and ****P < .0001

We performed flow cytometry analysis of Hs578T cells after stimulation of AXL by exogenous GAS6 for 30 min and 2 hours, in combination with BMS77607 treatment. We used two different extra-cellularly binding anti-AXL antibodies, namely Ab 154 and Ab 259/2. We analyzed the cell surface expression without cell membrane permeabilisation and total AXL abundance of Hs578T cell after cell membrane permeabilisation. Exogenous GAS6 stimulation resulted in a significant cell surface and total AXL depletion within 2 h to 50% or 60%. 0.5 μM BMS completely abolished the depletion of AXL from the cell surface (Fig. 5a-b). We show that internalization of AXL is suppressed by BMS and assumed that this is caused by inhibition of AXL ubiquitination (Fig. 5c-f).

### BMS777607 prevented ubiquitination of AXL after GAS6 stimulation

**Fig. 6:**
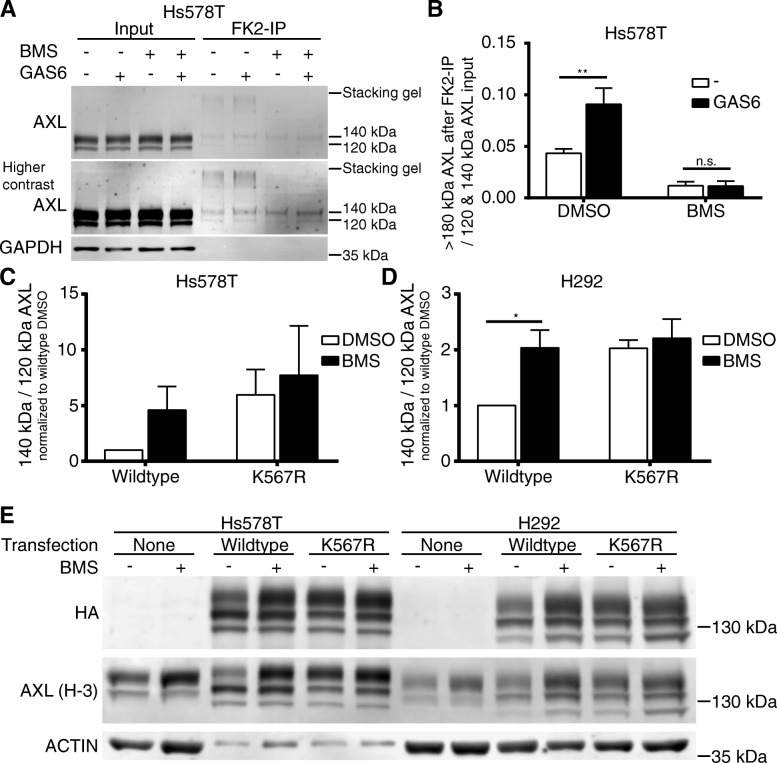
BMS777607 (BMS) treatment prevented ubiquitination of AXL. **a** Co-immunoprecipitation experiments of AXL and ubiquitin by using anti-ubiquitin antibody FK2 in Hs578T cell lysates after 1 h of BMS treatment and 250 ng/ml exogenous GAS6 ligand stimulation for 15 min are shown. The amount of precipitated AXL was subsequently determined by western blot. Ubiquitinated AXL was detectable as smear at high molecular weight in DMSO treated control lysates. (**b**) GAS6 stimulation increased the amount of ubiquitinated AXL significantly, in contrast to BMS, which blocked the ubiquitination completely. AXL western blot after treatment with 0.5 μM BMS of (**c**) Hs578T and (**d**) H292 cells which were transiently transfected with HA-tagged AXL gatekeeper mutant (K567R) and wildtype control. Exogenous AXL was quantified by anti-HA antibody and total AXL by a C-terminally binding anti-AXL antibody (H-3). 120 kDa AXL was used for normalization to consider transfection efficacy. BMS increased AXL 140kD/120kD protein ratio in wildtype overexpressing (**c**) Hs578T to fivefold and to twofold in (**d**) H292 cells. AXL gatekeeper mutant (K567R) is not responsive to BMS treatment, although exhibiting a similar AXL protein ratio compared to the wildtype transfected and BMS treated cells. (**e**) Representative western blots are displayed for Hs578T and H292 cells. Mean values and SEM of three independent experiments are shown. Differences with *P < .05, **P < .01, and ***P < .001 were considered statistically significant

We tested this hypothesis by performing co-immunoprecipitation experiments of AXL and ubiquitin by using anti-ubiquitin antibody FK2 (Fig. 6a). The amount of precipitated AXL was subsequently determined by western blot. Exogenous GAS6 ligand stimulation resulted in significant 2-fold increase of AXL ubiquitination. BMS completely abolished ubiquitination below the detection level (Fig. 6b). In summary we demonstrated that BMS inhibited ubiquitination of AXL after GAS6 binding and led to AXL cell surface accumulation.

### K567R gate keeper mutation prohibited the phosphorylation-dependent ubiquitination and subsequent internalization of AXL

BMS is an efficient AXL TKI, prohibiting AXL kinase phosphorylation [23, 25]. Finally, we investigated, if ubiquitin-dependent AXL internalization and subsequent lysosomal degradation depends on AXL kinase activity. Therefore, we introduced HA-tagged AXL gatekeeper mutants into Hs578T (Fig. 6c) and H292 cells (Fig. 6d). The K567R gate keeper mutation prohibits the binding of ATP to the hinge region of the kinase domain, which completely abolished the AXL phosphorylation. Analogous to previous experiments we analyzed the effect of 0.5 μM BMS on AXL abundance after 24 h of treatment [25]. The fully glycosylated 140 kDa AXL is localized at the cell surface and thereby accessible to GAS6. Consequently, only this 140 kDa AXL protein can be protected from GAS6-dependent degradation by BMS (Fig. 6e). We used the 120 kDa AXL protein as a normalization control for transfection efficacy in Hs587T and H292 cells (Fig. 6c-d). We observed that BMS increased the fully glycosylated 140 kDa AXL protein abundance in wildtype AXL transfected cells in similar fashion as the K567R gate keeper mutation. BMS treatment displayed no additional impact on the 140/120 kDa ratio in K567R gate keeper mutants. Consequently, we assume that AXL kinase activity and subsequent RTK phosphorylation is necessary for ligand-dependent receptor internalization and degradation. Blocking of kinase function by BMS resulted in phosphorylation prohibition, impaired internalization and subsequent cell surface accumulation, as observed in Fig. 1a-c. By using a plasmid based overexpression system without 3’and 5’UTR regions, we additionally excluded the impact of BMS on posttranscriptional regulation by miRNAs potentially binding to AXL mRNA untranslated regions.

In summary, we postulate that phosphorylation of AXL is a prerequisite of ubiquitin-dependent internalization and lysosomal degradation, which is completely abolished by BMS. The inhibition of AXL phosphorylation subsequently prevents ubiquitination and results in the accumulation of cell surface AXL via impaired internalization after GAS6 binding.

## Discussion

Our study identified elevated AXL cell surface expression after 24 h of BMS777607 AXL TKI treatment. The complex AXL RTK biology requires a better understanding of the underling mechanism for successful implementation of AXL targeting therapeutics, as therapy resistance still remains the major problem for targeted therapies. Therefore, we focused on mechanisms, by which AXL protein quantity is regulated, starting with transcriptional regulation [28, 29]. AXL expression is controlled by DNA methylation, histone acetylation and transcription factors, including SP1, AP-1 and HIFα, as reviewed by Gay et al. [4]. We excluded the impact of these transcriptional regulators on AXL protein abundance caused by BMS treatment as this would necessarily be caused by increased mRNA levels which could not be observed in our study (Fig. 1e-g). AXL expression is further regulated by post-transcriptional modifications. In dendritic cells, AXL expression is abundant, and in bone marrow-derived macrophages, AXL expression is minimal; however, there is essentially no difference in AXL mRNA copy number in these cells, suggesting a significant role for post-transcriptional or post-translational regulatory mechanisms of AXL expression [30]. Other studies have identified target sequences for microRNA (miRs) including miR-34 and miR-199a/b in the AXL 3′ untranslated region, with correlative findings confirming the effects of miRs on AXL expression [31, 32]. In our study we show that low μM BMS treatment does not influence AXL mRNA level. This was interesting, as we observed a minor increase of AXL protein levels already after 3 hours of BMS treatment in H292 cells (Fig. 3e-f). Using a plasmid based overexpression system without 3′ and 5′ UTR regions we exclude the impact of BMS on posttranscriptional regulation by miRNAs, potentially binding to AXL untranslated mRNA regions (Fig. 6c-d). Alternative splicing events have not been analyzed in this study, although three different splicing variants were described for the *axl* gene [33]. We haven’t observed a dramatic shift in AXL protein in western blot analysis after BMS treatment, but we cannot exclude a splice variant shift on mRNA level. Translational regulation of oncogenes play an important role in carcinogenesis [34]. Emerging evidence indicates that AXL expression may also be regulated at the translational level. A critical protein for translation initiation is eIF4E, which binds to the 5′ m7G cap of mRNA molecules and thus facilitates ribosomal recruitment [35]. In preliminary experiments we saw a slight, but significant, increase of eIF4B S422 phosphorylation in MDA-MB231 cells and increased ribosome-bound nascent chain puromycinylation in Hs578T and H292 cells after 24 h of low μM BMS treatment (data not shown). In contrast to that result we failed to validate a significant impact on translation by polysome-fractionation and subsequent RT-qPCR of bound AXL mRNA. Therefore, we cannot draw a clear picture, whether the translational machinery is significantly affected by BMS treatment leading to enhanced AXL protein enrichment. To become a functionally mature protein, important posttranscriptional modifications, including glycosylation and signal peptide cleavage, need to occur. Another type of AXL cleavage is commonly referred to as ‘ectodomain shedding’, in which the extracellular domain is cleaved from the cell membrane through actions of various matrix metalloproteinases and A Disintegrin and Metalloproteinase Domain (ADAM) family members, e.g. ADAM10 and ADAM17 [19]. We analyzed the impact of α-secretases and γ-secretases by combinatorial treatments of BMS or BB94 together with DAPT. BB94 blocks α-secretase activity and DAPT is a known inhibitor of γ –secretases. When taking reduction of receptor ectodomain shedding as a potential mechanism for 140 kDa AXL cell surface accumulation, then α-secretases have to be inhibited. Blocking of γ-secretases by DAPT treatment leads to the stabilization of the 55 kDa C-terminal fragment of AXL and causes no accumulation of the 140 kDa AXL protein. We could not prove an impact of BMS on the activity of α- secretases as shown in Fig. 4. Glycosylation is essential for maturation and function of membrane proteins regulating their routing, conformation and ligand binding. For example, inhibition of glycosylation sensitizes cancer cells that are resistant to EGFR targeted therapy to radiation. Tunicamycin inhibits N-acetylglucosamine (GlcNAc) transferase, which catalyzes the first step of protein N-glycosylation in the endoplasmic reticulum. Krishnamoorthy et al., 2013 could show that tunicamycin treatment of CAL62 cells led to AXL protein accumulation as a 100 Da protein in western blot, representing the core polypeptide, whereas the 140 and 120 kDa bands disappeared, indicating that both were N-glycosylated isoforms of AXL [12]. We have not focused on this aspect of posttranslational modification in the present study, but it is unlikely that glycosylation is impaired by BMS treatment as we could even observe an increase of the fully glycosylated 140 kD. Only exogenous over-expression of Ha-tagged-AXL showed the appearance of a potential non-glycosylated AXL protein at 110 kDa in western blot. Neither the 110 kDa band nor the 120 kDa band was significantly regulated by BMS treatment in contrast to the fully glycosylated 140 kDa band (Fig. 6e). This glycosylation of the endogenous or exogenous 140 kDa AXL protein is necessary for ligand accessibility and subsequent full activation of the kinase function [12]. Like for other RTKs, phosphorylation plays an essential role governing the activity and the fate of AXL. Upon GAS6 binding, AXL undergoes dimerization and Y698, Y702 and Y703 phosphorylation. Subsequently, three adjacent residues (Y779 and 821 and Y866) are phosphorylated, representing active docking sites for signal transduction. ATP binding is prohibited by a K567R gate keeper mutation resulting in an inactive kinase domain with loss of binding capability to adaptor proteins, like PI3K subunit p85 [16, 36]. Upon GAS6 binding AXL is activated, internalized and sorted to endosomes through endocytosis, which is clathrin- and dynamin-dependent. From endosomes, AXL can either be transported to the plasma membrane or delivered to lysosomes for degradation [37, 38]. We used chloroquine as a lysosomotropic agent that prevents endosomal acidification leading to inhibition of lysosomal enzymes. We could not show an additional impact by inhibition of lysosomal degradation with chloroquine on the abundance of AXL after BMS treatment (Fig. 4). AXL is also subject to ubiquitin-mediated proteasomal degradation. AXL degradation by proteasomes has been well demonstrated by Paolino et al.. Castias B-lineage lymphoma (CBL) is an E3 ubiquitin ligase, responsible for TAM family ubiquitination and degradation [18]. We could show in preliminary experiments that inhibition of the proteasom by MG132 does not lead to an accumulation of the 140 kDa AXL protein (data not shown). Phosphorylation of AXL tyrosine residues creates a docking site for recruiting CBL [13]. Alternatively, CBL may be recruited to AXL through its interaction with the adaptor protein GRB2 as shown for MET [39]. GRB2 is also implicated in the clathrin-mediated endocytosis of activated EGFR [40] and Pao-Chun et al. showed that GRB2 is binding to AXL after GAS6 ligand stimulation [41]. A similar GRB2/CBL-dependent internalization mechanism may exist for AXL as well, taking into account that Y821 is an essential tyrosine for binding of GRB2 [16]. AXL small molecule inhibitors prohibit receptor phosphorylation and prevent the binding of adaptor proteins. Among these, CBL leads to receptor downregulation by ubiquitination and successive degradation in the lysosomes. The activation-dependent downregulation is important for keeping a steady-state between active and non-active AXL modes. Here we demonstrated that kinase inhibition hinders the AXL activation-dependent downregulation. As a consequence, the AXL accumulates on the cell surface, where it potentially could be stimulated, once the administration of the inhibitor is stopped. Based on our data, we propose that AXL TKIs inhibit the phosphorylation on the CBL binding site, disrupting the interaction between AXL and CBL and thus restricting ubiquitination of AXL, which subsequently cannot be internalized and degraded in the lysosomes. Proper RTK downregulation is a crucial step of activity regulation, and if not tightly controlled, may cause oncogenic events. ATP binding of AXL is prohibited by a K567R gate keeper mutation resulting in an inactive kinase domain and shows analogous, but not additional effects like BMS treatment on AXL abundance (Fig. 6c-e). Here we report that impairment of downregulation takes place upon inhibiting the activity of AXL by TKIs. Although in the current case the receptor accumulates initially in an inactive state, enhanced phosphorylation of AXL might take place as well within 24–36 h, as shown for BMS by Baumann et al. and for R428 by Chen et al. [38, 42]. This might be relevant in vivo*,* where concentration gradients of drugs are common in tumors. Our findings could possibly translate into appalling consequences once the inhibitor drops below the inhibitory concentration in some cells within the tumor mass. To our knowledge, this is the first report to describe that targeting of the AXL by small molecule inhibitors leads to its cell surface accumulation by potential interference with a downregulation-associated mechanism. This phenomenon might represent clinically relevant aspect to be considered when following AXL inhibition in clinical setups. Additionally, the release of the soluble 85 kDa N-terminal fragment (sAXL) might be used as biomarker and campaign diagnostic tool as shown for early hepatocellular carcinoma (HCC) and malignant peripheral nerve sheath tumors (MPNST) [20, 21]. We demonstrated that the 55 kDa C-terminal fragment, which is generated as the sAXL is shedded by ADAM10 and 17, was accumulating after BMS treatment in Hs578T (Fig. 4). Consequently, sAXL might be increased in serum of AXL TKI treated patents [19]. Beside kinase inhibition with TKIs or decoy receptors also receptor downregulation is a common mode of action for AXL targeting therapies. This was shown for antagonistic monoclonal antibody targeting AXL [43], 17-Allylamino-17-demethoxygeldanamycin [12] and yuanhuadine [11]. As a linkage of kinase inhibition and receptor degradation, proteolysis targeting chimera (PROTAC) technology could be used as a powerful tool for AXL targeting therapy. Burselm et al. could demonstrate that PROTACs are capable of inducing the degradation of active EGFR, HER2, and c-Met. In most cases, PROTACs capable of degradation inhibit downstream signaling and cell proliferation at lower concentrations than similar TKIs without linked degradation machinery recruiting unit. Furthermore, degradation provides a more sustained reduction in signaling, as evidenced by the reduction in kinome rewiring, as observed previously with EGFR, HER2, and c-Met inhibitors, as well as the sustained duration of response even after washout [44]. Based on our study, we would suggest a PROTAC-based strategy for AXL inhibition to achieve a sustained inhibition and depletion of AXL. This might enhance the efficacy of targeted AXL therapies in the clinics.

## Conclusion

AXL tyrosine kinase inhibitors (TKIs) are currently under clinical evaluation. We observed by Western blot and flow cytometry analysis that AXL TKI BMS increases AXL protein levels after 24 h of treatment. We demonstrate that AXL kinase activity and subsequent RTK phosphorylation is necessary for GAS6-dependent receptor internalization and degradation. Blocking of kinase function by BMS results in phosphorylation prohibition, impaired internalization and subsequent cell surface accumulation. Our data suggest careful consideration of anti-AXL clinical protocols because feedback loops and resistance formation might countervail targeted AXL therapy. An alternative strategy to circumvent feedback loops for AXL targeting therapies may exist in linkage of AXL TKIs to a degradation machinery recruiting unit (PROTACs). This might result in a sustained inhibition and depletion of the AXL from tumor cell surface and enhance the efficacy of targeted anti-AXL therapies in the clinics.

## Data Availability

Data and materials are available from the corresponding author on reasonable request.

## Abbreviations

3D: three-dimensional
ADAM: A Disintegrin and Metalloproteinase Domain
AKT: RAC-α serine/threonine protein kinase
AP-1: AP-1 transcription factor subunit
AXL: AXL receptor tyrosine kinase
BMS (BMS777607): N-(4-(2-Amino-3-chloropyridin-4-yloxy)-3-fluorophenyl)-4-ethoxy-1-(4-fluorophenyl)-2-oxo-1,2-dihydropyridine-3-carboxamide
CBL: Castias B-lineage lymphoma
CHX: Cycloheximide
c-Met: MET proto-oncogene, receptor tyrosine kinase
CQ: Chloroquine
DAPT: N-[N-(3,5-Difluorophenacetyl)-L-alanyl]-S-phenylglycine t-butyl ester
DMSO: Dimethyl sulfoxide
EGFR: Epidermal growth factor receptor
eIF4B: eukaryotic translation initiation factor 4B
eIF4E: eukaryotic translation initiation factor 4E
ERK: Extracellular signal-regulated kinase
FCS: Fetal calf serum
GAS6: Growth arrest specific gene 6
GRB2: Growth factor receptor bound protein 2
HER2: erb-b2 receptor tyrosine kinase
HIFα: Hypoxia inducible factor 1 subunit alpha
MG132: Benzyl N-[(2S)-4-methyl-1-[[(2S)-4-methyl-1-[[(2S)-4-methyl-1-oxopentan-2-yl]amino]-1-oxopentan-2-yl]amino]-1-oxopentan-2-yl]carbamate
miRs: microRNA
NF-κB: the nuclear factor-kappa B
p38: p38 mitogen-activated protein kinase
PI3K: Phosphatidylinositide 3-kinase
PROTAC: Proteolysis targeting chimera
RTK: Receptor tyrosine kinase
RT-qPCR: Reverse transcription-quantitative PCR
SEM: Standard error of the mean
SP1: Sp1 transcription factor
STAT: Signal transducer and activator of transcription signaling
TKIs: Tyrosine kinase inhibitors
